# Supplementary material for: A genome editing approach to study cancer stem cells in human tumors
Source: EMBO Mol Med. 2017 May 3;9(7):869–79. doi: 10.15252/emmm.201707550 (PMC5494503; doi:10.15252/emmm.201707550)
Supplement: Supplementary file 1 — Appendix [file EMMM-9-869-s001.pdf]

APPENDIX

Report

A genome editing approach to study cancer stem cells in human tumors

Carme Cortina*, Gemma Turon*, Diana Stork, Xavier Hernando-Momblona, Marta Sevillano, Mònica Aguilera, Sébastien Tosi, Anna Merlos-Suarez, Camille Stephan-Otto Attolini, Elena Sancho and Eduard Batlle

TABLE OF CONTENTS

APPENDIX SUPPLEMENTARY TABLES

Page

Appendix Table S1. CRC Driver Mutations 2

Appendix Table S2. CRISPR/Cas9 knock-in efficiencies 3

Appendix Table S3. Summary results of exome sequencing comparing PDO#7 parental versus LGR5-EGFP Clone #14 4

Appendix Table S4. sgRNA most likely offtargets in exons 5

Appendix Table S5. Exact P-values 6

APPENDIX SUPPLEMENTARY FIGURES

Appendix Figure S1. Examples of genotyping PCR and southern-blot to assess LGR5-EGFP cassette integration 7

APPENDIX SUPPLEMENTARY METHODS 9

Appendix Table S1. CRC Driver Mutations

PDO	Stage	Grade	Site	WNT pathway	KRAS pathway	PI3K pathway	TP53/ ATM	TGF-beta Pathway
**#6**	II	G1	Right Colon	APC FS (-522) /+	KRAS (A146V/+)	WT	WT	WT
**#7**	IV	G3	Caecum	APC STOP (R787*)/STOP (K1438*)	KRAS (G13/+)	WT	ATM (V182L / N1983S)	SMAD4 (L536R /L536R)

Appendix Table S2. CRISPR/Cas9 knock-in efficiencies

Knocked-in locus	Tumoroid	% Positive cells 20 d post-nucleofection (3)	Single cell on-target clones (4)	clones without off-target integrations (5)
**Lgr5$^{LF2A-EGFP}$**	PDO#7	4.95%	85.3% (35/41)	41.7% (5/12)
	PDO#6	6.02%	76.3% (42/55)	84.6% (11/13)
**Ki67^{tagRFP2}**	Lgr5$^{LF2A-EGFP}$ #1	0.13%	70.8%	100% (17/ 17)
**AAVS1^{LBLT}**	PDO#7	22.3%	47.8%	20% (2/10)
**Lgr5^{CreERT2}**	AAVS1^{LBLT}#1	10.7% (at d2)	1.78%	50% (4/8)

Appendix Table S2. CRISPR/Cas9 knock in efficiencies for the different targeting vectors. Column 3 indicates the percentage of cells positive for reporter genes 20 days after nucleofection (respectively for each targeting: EGFP+, TagRFP2+ and mTagBFP2+). The LGR5-CreERT2 targeting vector lacks fluorescent marker and therefore single cell clones were derived directly from the pool of iRFP+ cells sorted at day 2 post-nucleofection. Column 4 indicates the proportion of single cell derived clones from the sorted pool at day 20 that scored positive by integration PCR. Column 5 shows the number of integrated clones without off-targets integrations assessed by southern blot (See Supplementary Figure 1 for examples of genotyping).

Appendix Table S3. Summary results of exome sequencing comparing PDO#7 parental versus LGR5-EGFP Clone #1

	**Predicted sgRNA most-likely off-targets in exons**	**CRC driver mutations**	***de novo* missense or non-sense**	***de novo* mutations with predicted high impact (genes)**
Mutect	None mutated	All present (APC, KRAS, SMAD4, ATM)	64	3 (EIF2AK2, PPFIBP2, KIAA0101)
VarScan	None mutated	All present (APC, KRAS, SMAD4, ATM)	70	6 (CCRN4L, SEMA5A, HOOK3, PRSS3, GLE1, PPFIBP2)
Common	None mutated	All present (APC, KRAS, SMAD4, ATM)	17	1 (PPFIBP2)

Appendix Table S3: Exome sequencing results of PDO7 parental vs PDO7 LGR5-EGFP#1 using two different Variant Caller softwares. We have used two different softwares, Mutect and VarScan, to analyze the same exome sequencing data to try to reduce the number of detected false positives. Both analysis indicate that our clone maintains the mutations the CRC driver mutations and do not has mutations in the CRISPR/Cas9 guide most predicted offtarget sites. The number of de novo missense or non-sense mutations detected by the two algorithms was very similar but only a subset of 17 variants were common to the two analysis suggesting that the rest were false positives.

Appendix Table S4. sgRNA most likely offtargets in exons

Sequence	Mismatches	Gene	Location	Clone #1
TGTCTCTGAATAGTAGGTGA GGG	4MMs [8:10:13:16]	GPR3 (NM_005281)	Exon 2 bp 521-543 aa 141-167	wt
CACCCCTAATTAATATGTGA GAG	4MMs [1:2:3:5]	SH2D4B (NM_207372)	Exon 7 Bp 3748- 3771 3'UTR	wt
TTTTTCTATTCAATATGTGAA AG	4MMs [2:4:9:11]	MT1B (NM_005947)	Exon 3 Bp 325-357 3'UTR	wt
TATCTCTAATGAAATTGTGAA AG	4MMs [2:11:14:15]	PRND (NM_012409)	Exon 2 Bp 1118-1140 3'UTR	wt
TATCTATAAATAATTTGTGAA GG	4MMs [2:6:10:15]	USF3 (NM_00100989 9)	Exon7 bp 13257-13280 3'UTR	wt

Appendix Table S5. Exact p-values

Figure	Number of experiments (n)	Statistical test	Specific p-values
Fig 2G	N= 4 wells per condition. Tumor cells for all experimental points were purified from the same xenograft.	Student T-test	Number = 0.003047
			Size= 0.020257
Fig 2I	N= 9 independent xenografts per condition. All tumor cells inoculated were isolated from the same primary xenograft.	Log-Rank (Mantel-Cox) test	1000 injected cells = 0.000027
			200 injected cells =0.000014
Fig 3J MUC2	N d14 = 3 xenografts<br>N d28 = 8 xenografts<br>N d56= 8 xenografts	Generalized linear model with mixed effects and binomial family. the function glmer from the R package "lme4" was used to fit the model, compute p-values and estimate fixed effects	14 days vs 28 days = 0.00000121
			14 days vs 56 days = 0.000528
Fig 3J KRT20	N d14 = 3 xenografts<br>N d28 = 8 xenografts<br>N d56= 8 xenografts	Generalized linear model with mixed effects and binomial family. The function glmer from the R package "lme4" was used to fit the model, compute p-values and estimate fixed effects	14 days vs 28 days = 0.000053
			14 days vs 56 days= 0.0000000234
			28 days vs 56 days= 0.000772
Fig EV1J	N= 4 wells per condition. Tumor cells for all experimental points were purified from the same xenograft	Student T-test	Number = 0.001739
			Size= 0.002146
Fig EV3F	N= 4 wells per condition. Tumor cells for all experimental points were purified from the same xenograft	Student T-test	Number = 0.000043
			Size = 0.000119
Fig EV3H	N= 9 independent xenografts per condition. All tumor cells inoculated were isolated from the same primary xenograft.	Log-Rank (Mantel-Cox) test	1000 injected cells = 0.000213
			200 injected cells =0.0391

APPENDIX FIGURE S1

A

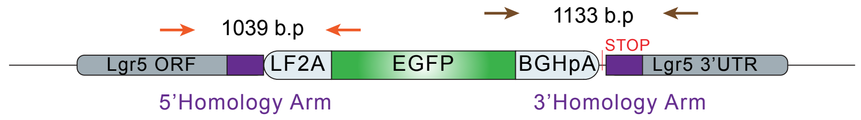

B

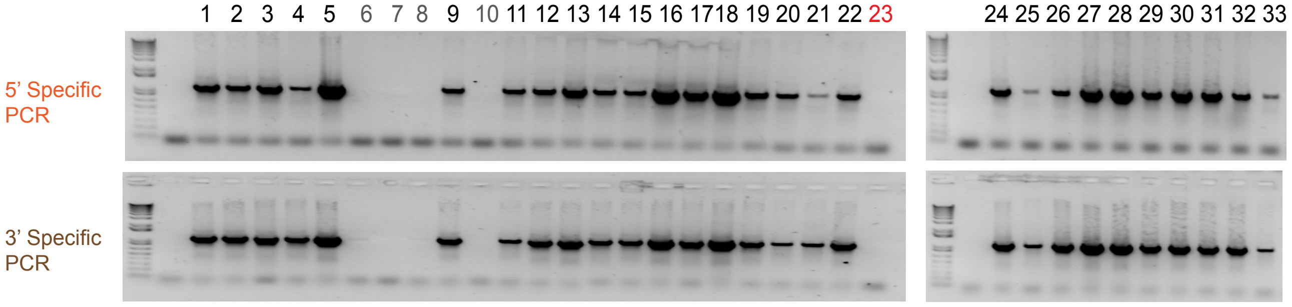

C

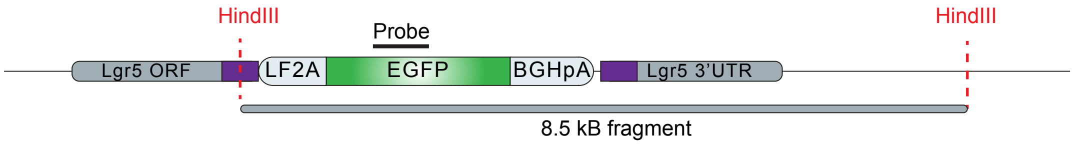

D

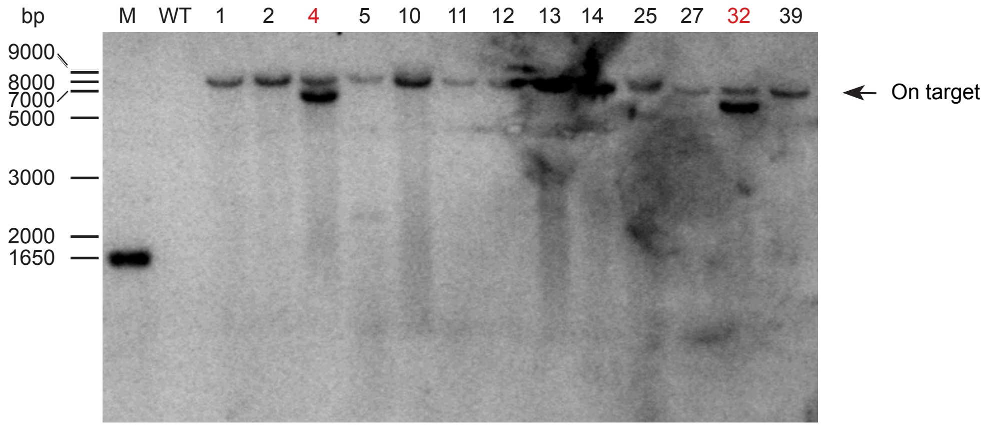

Appendix Figure S1. Examples of genotyping PCR and southern-blot to assess LGR5-EGFP cassette integration. **A.** Scheme showing primer location for the specific integrations PCRs (in orange, 5’ specific; in brown, 3’ specific) **B**. 5’-specific, 3’-specific genotyping PCRs of PDO#7-derived clones. **C.** Scheme of the southern blot design for the LGR5-EGFP locus. **D.** Southern Blot using EGFP probes in PDO#7-derived clones. Black numbers represent positive clones; red numbers represent clones with more than one integration site.

APPENDIX SUPPLEMENTARY METHODS

Donor plasmid construction

750 bp (LGR5, KI67 and AAVS1 constructs) of 5' homology arm (HA) and 3' homology arm were amplified from PDO7 gDNA or synthetized by gene synthesis (Thermo Fisher) and cloned in pShuttle or pDONR vectors. LF2A-EGFP-BGHpA, linker-tagRFP2, LF2A-CreERT2-BGHpA, Lox-tagBFP2-3xpA-Lox-tdTomato-BGHpA insertion cassettes were generated by gene synthesis (Thermo Fisher) and cloned in the 5'HA-3'HA previously engineered pShuttle or pDONR vectors.

sgRNA design

Small guide RNAs were designed using the http:// crispr.mit.edu web tool. To select for the most suitable sgRNAs, we applied the following criteria: i. localization of the sgRNA as near as possible to the desired site of insertion to maximize homologous recombination efficiency, ii. Cas9-mediated double strand break downstream of STOP codon to prevent NHEJ-induced indels in the ORF, iii. guides selected to anneal at the intersection between the 5' homology arm and 3' homology arm so that the donor plasmid is protected from Cas9 cut, iv. minimum off-target score according to http://crispr.mit.edu and maximum Doench activity score (1). sgRNAs used to modify PDOs were as follows: LGR5 (TGTCTCTAATTAATATGTGA), KI67 (TTTGACAGAAAAATCGAACT), AAVS1 (GTCCCTAGTGGCCCCACTGT).

px330-IRFP Cas9 plasmid construction

Px330 Cas9 plasmid from Feng Zhang's laboratory was obtained from addgene (ref. 42230) and was modified by the introduction of a SV40promoter-IRFP expression cassette downstream of Cas9 by FseI - EcoRI. In addition the BbsI site of IRFP was silenced by site-directed mutagenesis. SgRNAs were cloned in px330-IRFP as described in http://www.genome-engineering.org/crispr/wp-content/uploads/2014/05/CRISPR-Reagent-Description-Rev20140509.pdf

CRISPR/Cas9 knock-in generation in PDOS

Nucleofection

For PDO nucleofection, 2 million of single-cell trypsinized PDO cells were nucleofected with 7 µg of donor plasmid and 2 µg of px330-IRFP Cas9 corresponding plasmids using Lonza nucleofector kit V (VVCA-1003) and program A-32 in an Amaxa-II nucleofector following manufacturer protocol.

FACS strategy and generation of single cell-derived organoids

Nucleofected cells were embedded in BME2 drops and cultured for 2-3 days in PDO medium. We then isolated cells that were IRFP+ (i.e. had incorporated the targeting vector) by FACS and cultured them in 3D for about 18-20 days. For the AAVS1 lineage tracing cassette, IRFP+/tagBFP2+ double positive cells were sorted. After 18-20 days in culture, we observed the emergence of a cell population that expressed the marker gene of interest suggesting that some cells had integrated reporter vector. We then selected the cell population positive for expression of EGFP (LGR5-LF2A-EGFP), tagRFP2 (KI67-tagRFP2) or tagBFP2 (AAVS1-LSL-TOM), which was at the same time negative for IRFP. Cells were seeded in a 96-well format to derive single-cell clones. Wells with more than one cell per drop were discarded. LGR5-LF2A-CreERT2 targeted cells were plated in single cell format directly after IRFP+ cell sorting.

Specific genotyping PCRs

Single-cell derived clones were lysed in buffer consisting of 10 mM Tris, 1 mM EDTA, 1 % Tween 20 and 0.4 mg/ml proteinase K for 1 h at 55 °C. The lysate was directly used in the specific integration PCR. For the 5’ specific integration PCR a forward primer upstream of the 5’ homology arm and a reverse primer at the beginning of the inserted cassette were used. Similarly, for the 3’ specific integration PCR a forward primer at the end of the inserted cassette and a reverse primer downstream of the 3’ homology arm were used. The PCR conditions were as follows: DNA Polymerase (BioTools #10012-4103) 95 ºC 2 min x38 (95 ºC 30 s – 55 ºC 30 s – 72 ºC 1:30 min) 72 ºC 5 min - hold 16 ºC. Used primer sequences are shown in table 1.

Table 1: Primers used for the specific integration PCR

Locus-insertion		Primer
LGR5-LF2A-EGFP	5’ specific	F: GTTTTTTGTCCATTTTTGCTTCAG
		R: ACCACCCCGGTGAACAGC
	3’ specific	F: TCGGCATGGACGAGCTGTACAAG
		R: AGTGGAATTCCCTTCTGAGCTTTG
LGR5-LF2A-CreERT2	5’ specific	F: GTTTTTTGTCCATTTTTGCTTCAG
		R: CGAACATCTTCAGGTTCTGCGGG
	3’ specific	F: TTCCTTGCAAAAGTATTACATCAC
		R: AGTGGAATTCCCTTCTGAGCTTTG

KI67-tagRFP2	5’ specific	F: CCGTCGAGTCTTACAATAAAACG
		R: TCAATTAAGTTTGTGCCCCAG
	3’ specific	F: ATGGTGTCTAAGGGCGAAGAG
		R: TACAGAGTACTGGTGTCACTTCCTG
AAVS1-LSL-TOM	5’ specific	F: GGACCACTTTGAGCTCTACTGG
		R: GGGCCATTTACCGTAAGTTATGTA
	3’ specific	F: GCATTGTCTGAGTAGGTGTCATTC
		R: ATGAGATGGTGGACGAGGAAGG

Southern blot

Clones that were correctly targeted, based on 5’ and 3’ specific integration PCRs, were further checked for off-target cassette insertions by southern blot. Genomic DNA was extracted using the GenElute Mammalian Genomic DNA Miniprep Kit (Sigma G1N70-1KT). 10 ug of genomic DNA were digested overnight with the appropriate restriction enzyme (table 2) and separated on a 0.8 % agarose gel. DNA fragments were transferred by capillarity to a Hybond-N+ membrane (GE Healthcare RPN303B) overnight. Probes were generated by PCR (protocol as described in the previous section, primers in table 2) and radioactively labelled with α-[^{32}P]dCTP using the MegaPrime labelling kit (GE Healthcare RPN1604). Hybridization with the probe was carried out overnight at 60 ºC. Probes were detected using a Phosphoimager plate.

Table 2. Primers used for southern blot probe generation and restriction enzymes used to digest each construct.

Locus-insertion	Primer	Restriction enzyme
LGR5-LF2A-EGFP	F: CCACCATGGTGAGCAAGGGCGAGG<br>R: TTACTTGTACAGCTCGTCCATGCC	HindIII
LGR5-LF2A-CreERT2	F: ATGGACATGTTCAGGGATCGCCAGG<br>R: GATTACGTATATCCTGGCAGCGATC	PvuII
AAVS1-LSL-TOM	F: GGGCATGGCACCGGCAGCACC<br>R: CCTACTTGTACAGCTCGTCCATGCC	PvuII

Mouse studies

NOD-SCID (NOD.CB17-Prkdcscid/J) female mice were purchased at Janvier Labs. They were used at 6 weeks of age at the beggining of the experiments, which took

place during a maximum of 21 weeks until the animals showed signs of distress. Animals were kept in Barcelona Science Park Animal Facility (SEA-PCB). All mouse experiments were approved by the Animal Care and Use Committee of Barcelona Science Park (CEEA-PCB) and the Catalan government. 150,000 cells (PDO#7) or 2 million cells (PDO#6) in a format of 5 to 7-days grown organoids were injected subcutaneously into NOD/SCID female mice in 50% BME2-HBSS with a maximum of 4 xenografts per animal. Tumor volume was measured with manual calipers and using the formula (length x width x height)/2. Mice were sacrificed when tumors reached a maximum of 300 mm^3, the animal displayed ulceration in one of the xenografts or showed symptoms of distress. Xenografts were resected and disaggregated as previously described in Merlos-Suárez et al, 2011 (3). Human epithelial cells from disaggregated xenografts were stained with hEPCAM-PeCy7 1/150 (eBioScience 25-9326-42) or hEPCAM-APC-Vio770 1/75 (Miltenyl Biotec 130-101-161). DAPI 1µg/ml was added to distinguish alive/dead cells. The cell suspension was analyzed with a BD Aria Fusion FACS or Aria FACS according to the following criteria: debris was discarded by FSC-A/ SSC-A gating, aggregates by FSC-A/FSC-W gating, alive cells by DAPI negative signal (VioF ultraviolet or violet channel 360 V), human EPCAM positive cells were selected by Green A (414 V) or Red A channels (500 V). Cells that were positive for the criteria mentioned above were analyzed for EGFP (Blue B channel, 500 V), TdTomato and tagRFP2 (Green D channel, 500 V)

Tumor initiation assays

Viable (DAPI-) human EPCAM+ single cells from disaggregated xenografts were sorted according to their EGFP positivity and subsequently transplanted into new recipient mice at limiting dilutions. We injected 200 or 1000 cells per flank in 100 µl of BME2-HBSS 50 % - 50 % (n=12). Tumor volume was measured twice a week. When a xenograft reached 300 mm^3, it was resected from the animal. The experiment finished when all xenografts were grown or when the animals were 21 weeks old. Differences were assessed with Log-Rank (Mantel Cox) test.

Organoid Formation assays

Human EPCAM + alive single cells from disaggregated xenografts were isolated by FACS according to their EGFP positivity as described before and seeded *in vitro* in 25 µl BME2 drops containing 1000 cells/drop (n=4) per condition. Then, plates were scanned with a ScanR inverted microscope at day 1 post-seeding to quantify the exact number of cells seeded per drop and at experimental endpoints (day 14 post-seeding). Full drops were scanned taking overlapping pictures at 4x magnification and at 8

different z-stacks with a separation of 200 µm among them. Z-stacks of each field of view were projected in a single image and the full drop was digitally reconstructed by stitching the different image projections using an Image J custom-made macro developed for this purpose. Total number and mean size of cells (i.e. any object with a diameter larger than 5 µm) or organoids (diameter larger than 400 µm) were counted. Differences were assessed with Student´s t-test.

Dapi content analysis

A minimum of 50,000 cells for each population of interest were sorted with a BD Aria Fusion FACS as described and fixed with EdU-cell cycle kit fixative for 15 min (Life Technologies, C10424). DAPI (1µg/ml) was incubated for 1 hour and cells were subsequently analyzed with a BD Aria FACS gated from EPCAM-positive. Cell cycle stages were determined using WinCycle v4.0

Real-time qPCR

1000 cells from either EPCAM-stained disaggregated xenografts or from *in vitro* cultured PDOs fulfilling the fluorescence criteria stated for each experiment were sorted directly in 45 µl of picoprofile lysis buffer. RNA and cDNA was extracted and amplified as previously described in González-Roca et al, 2010 (4). Concentration of the obtained cDNA was measured with a Nanodrop. 5 ng of cDNA were used per each real-time qPCR well. Real-time qPCRs were performed with TaqMan assays and TaqMan Universal PCR Master Mix (Applied Biosystems 4369016) or SYBR-Green PCR Master Mix (Applied Biosystems 4309155) in triplicates following manufacturer's instructions. The following TaqMan assays (ThermoFischer) were used:

AURKB (Hs00177782_m1), CDKN1A (Hs_00355782_m1), CHGA (Hs00900370_m1) CreERT2 (custom), EFNB2 (Hs00187950_m1), EGFP (Mr04097229_mr), EPHB2 (Hs00362096_m1), FOXM1 (Hs00153543_m1), KRT17 (Hs00356958_m1), KRT20 (Hs_00300643_m1), LGR5 (Hs_00173664_m1), MKI67 (Hs_01032443_m1), MUC2 (Hs_03005094_m1), MYB (Hs00193527_m1), MYC (Hs00905030_m1), OLFM4 (Hs_00197437_m1), SMOC2 (Hs0159663_m1), TdTomato (custom), UBE2C (Hs00964100_g1). All real-time qPCRs were normalized to B2M (Hs_99999907_m1) and PPIA (Hs_99999904_m1) expression.

The following primers for SYBR Green detection were used:

SI (F: 5’-TCCAGCTACTACTCGTGTGAC-3’ / R: 5’-CCCTCTGTTGGGAATTGTTCTG-3’) FABP1 (F: 5’AAGACAGTGGTTCAGTTGGAAG-3’ / R: 5’-TGAGTTCGGTCACAGACTTGAT-3’).

Microarray analysis

Affymetrix array data were normalized using RMA background correction and summarization (5) as implemented in the "affyPLM" package (6) from R. Annotations for the Human PrimeView array version na32 were downloaded from Affymetrix (7). Technical metrics were computed for each sample as described in (8). All samples passed quality controls. A linear model was fitted to find differentially expressed genes between conditions of interest with technical metrics (pm iqr, pm median, RNA degradation) and biological replicates as covariates. The "lmFit" function from the "limma" package (9) was used for fitting the model. Microarray data for PDO7-Lgr5-EGFP high vs. negative cells is available at GSE92960. Microarray data for PDO7-Lgr5-EGFP/Ki67-tagRFP2 is available at GSE92961. Gene set enrichment analysis (GSEA) as implemented in (10) was performed on ranked lists with all genes in the array. For each gene the fold change of the most variable probe was used as representative. We also ran GSEA on previously published gene sets (3, 11).
We also downloaded the expression matrix from GEO accession GSE52813 (12) and performed differential expression analysis between groups “Lgr5hi_Ki67hi” and “Lgr5hi_Ki67low”. We defined signatures by filtering genes with a p-value lower than 0.05 and fold change larger (smaller) than 1.5 (-1.5).

Whole Exome Sequencing

The library preparation for capturing of selected DNA regions (Agilent Human All Exon 50Mb v5, Agilent) was performed according to Agilent’s SureSelect protocol v1.8 for Illumina paired-end sequencing. In brief, 3.0 µg of genomic DNA was sheared on a Covaris™ E210 instrument (Covaris). The fragment size (150-300 bp) and the quantity were confirmed with an Agilent 2100 Bioanalyzer 7500 chip. Agilent paired-end adaptors were ligated to fragmented DNA followed by PCR amplification (6 cycles) with SureSelect Primer and SureSelect Pre-capture Reverse PCR primers and hybridized for 24 h at 65 ºC. The hybridization eluate was PCR amplified (16 cycles) in order to add the index tags using SureSelectXT Indexes for Illumina. The final library quality, control of the size and the concentration were determined on an Agilent 2100 Bioanalyzer 7500 chip.

Each library was sequenced on an Illumina HiSeq 2000 instrument in a fraction of a sequencing lane, HiSeq PE Cluster Kit v3, following the manufacturer’s protocol, with a paired end run of 2x101 bp, with coverage ofat least 96 % of the target region covered by at least 10 sequencing reads (C10 >96 %). Image analysis, base calling and quality

scoring of the run were processed using the manufacturer's software Real Time Analysis (RTA 1.13.48, HCS 1.5.15.1) and followed by generation of FASTQ sequence files by CASAVA. Samples were aligned to the human genome version hg19 with bwa (13) using the mem algorithm. Somatic mutations were detected using Mutect2 with default parameters (14) and compared to those obtained by VarScan2 (15). Sequences have been deposited at ENA (PRJEB18738). The control sample was previously submitted to ENA with accession number PRJEB7932 (Organoid P8) (2).

Cell cycle analysis

Mice bearing subcutaneous xenografts were injected with EdU 80 mg/Kg 3 h before their sacrifice. Xenografts were resected, disaggregated and stained with human-EPCAM-FITC (Ab112067, dilution 1/75) and DAPI at 1µg/ml as described above. 100,000 DAPI negative cells were sorted, fixed with EdU-cell cycle kit fixative for 15 min and subsequently stained with click-iT Alexa-647 reagent and DAPI following manufacturer's protocol (Life Technologies, C10424). Cells were subsequently sorted in a FACS Aria according to their Edu-Alexa647 signal (red A channel 500 V) and their DNA content (vioF channel 340 V) gated from EPCAM-positive cells which were tagRFP2 positive or tagRFP2 negative.

Immunostaining and confocal imaging of *in vitro* cultured PDOs

PDOs were seeded and grown for 2 weeks without trypsinization and then harvested using Matrisperse Cell Recovery Solution (Corning, 354253). PDOs were seeded at 100.000 cells per well in microscopy chamber slides in thin layers of BME (20 uL per chamber). For alive imaging, organoids were 30 min incubated at 37 ºC with Hoechst 33342 1:1000 (Molecular Probes, R37605) or RedDot1 1:200 (Biotium, 40060). For immunocytostainings, samples were fixed with 4 % paraformaldehyde for 15 min and blocked with 20 mM glycine for 20 min. Permeabilization was achieved with 0.5 % Triton X-100 for 30 min at room temperature, then a second blocking step with 1 % BSA for 1 h at room temperature was performed. Samples were incubated with primary antibodies overnight at 4 ºC in a dark chamber followed by the incubation with the correspondent secondary antibody for 1 h at room temperature in the dark. DAPI (1:5000) was added after secondary antibody staining for 15 min at room temperature. Washes were performed in between each step with PBS. Primary antibodies against KRT20 (mouse, Dako, M7018), MUC2 (mouse, AbCam, Ab118964) and GFP (goat, Abcam, ab6673) were used at 1:100 dilution. Secondary antibodies donkey anti-goat and anti-mouse conjugated to Alexa-488(DαG) and Alexa-647 (DαR, DαM) (Life

Technologies, A21447, A31571 respectively) were used at 1:200 dilution. Images were taken with LEICA SP5 confocal microscope.

Immunostaining on paraffin sections

Whole xenografts were cut in halves and fixed in formalin o/n. The fixative was removed and tissue was washed for 2 h in PBS before paraffin embedding. Immunostainings were performed on 4 um tissue sections according to standard procedures. Briefly, for immunohistochemistry (IHC) antigen retrieval was carried out with boiling Tris-EDTA buffer for 20 min, then samples were blocked with Peroxidase-Blocking Solution (Dako: S202386) for 10 min at room temperature and incubated with primary antibody against TdTomato (rabbit, Rockland, 600-401-379) 1:200 overnight. Bridging was performed with a Goat anti-Rabbit antibody 1:200 (Jackson Immunologic, 111-005-003) for 1 h at room temperature. Secondary antibody Anti-goat-HRP (Immunoresearch, DPVG55HRP) 1:200 was added for 30 min at room temperature. Slides were developed using DAB (Dako, K346811) and counterstained with hematoxylin prior to mounting with DPX (Panreac, 255254.1608). Washes were performed in between steps with wash buffer (Dako, K800721). Serial sections were used for hematoxylin-eosin staining according to standard procedures. For immunofluorescence the applied protocol was the same as for IHC with the following primary antibodies: TdTomato or RFP (rabbit, Rockland, 600-401-379) 1:200, KRT20 (mouse, Dako, M7018), KI67 (mouse, Novocastra, 6004992), MUC2 (mouse, AbCam, Ab118964) and secondary antibodies: donkey anti-goat conjugated to Alexa-488 (Life Technologies A21447) and donkey anti-mouse conjugated to Alexa-568 (Life Technologies A10037). Bridging for TdTomato antibody was performed with Goat anti-Rabbit antibody 1:200 (Jackson Immunologic, 111-005-003) for 1 h at room temperature. DAPI was added at 1:2500 after secondary antibody incubation and slides were mounted with Fluorescent mounting media (Dako 53023).

Quantification of the lineage tracing clone number and area

All images for lineage tracing quantification were acquired using a NanoZoomer-2.0 HT C9600series scanner (Hamamatsu, Photonics, France) with the 20X objective and coupled to a mercury lamp unit L11600-05 and using NDP.scan2.5 software U10074-03 (Hamamatsu, Photonics, France). All brightfield images were visualized with a gamma correction set at 1.8 with the NDP.view 2U123888-01 software (Hamamatsu).

The total epithelial area was estimated from hematoxylin-eosin stained sections of the xenografts by manually training a machine learning pixel classifier (Trainable Weka Segmentation, Fiji ImageJ distribution (16). Local colorimetric, textural and structural

features were included in the feature set used by the classifier. To reduce the level of details and speed up the operation the images were downscaled by a factor 8 prior to classification.

TdTomato IHC scans were downsampled by a factor 4 and analyzed by manually training a machine learning pixel classifier (Ilastik software [17]). This time, only intensity based local features were included and the clones were identified as TdTomato+ 2D connected particles in the resulting classification masks.

Clones at 4 days were counted manually rather than with Ilastik as the relatively weak levels of staining imposed severe limitations to image analysis. We also noticed that secondary tumors generated after passaging using a trocher displayed non-labeled cells intermingled within clones. This issue complicated clones identification as they often appeared fragmented, which biased their consecutive count and area estimation. To limit this problem we applied a post-processing step aiming at clustering neighbor 2D connected particles in the classification masks. It was implemented as a custom ImageJ macro (ADM, IRB Barcelona) leveraging morphological closing by a disk of user defined size (set to 8 pixels for all conditions).

The results of the analysis are summarized in Table 3:

Table 3: Number of xenografts and TdTomato clones assessed per timepoint.

Time (days)	Number of tumors	Number of clones
4	4	878
14	5	2424
28	8	6940
56	8	333

In order to estimate levels of background noise in area quantification only clones from day 4 were used. The logarithms of the areas were clustered using the Mclust function from the "mclust" package (18) in the R statistical environment (19), finding 2 clusters through the Bayesian Information Criterium. The cluster with lower mean was considered to be background. Clones with area lower than the 25 percentile of the remaining cells were filtered out from the dataset. Since most clones from day 4 consist of a single cell, the median of these areas was used as cell size for each experiment. Clone sizes were defined as consisting of 1 cell, 2, 3 or 4, 5 to 10, 11 to 100 and more than 100 cells.

Quantification of differentiation and proliferation markers within the clones

Immunofluorescent images were acquired at 20X with the Nanozoomer scanner. DAPI signal of all the samples was acquired with the DAPI350 filter with an exposure time of 28ms and a gain of 3. Tom-Alexa 488 and eGFP-Alexa 488 signal of all the samples was acquired with the FITC filter with an exposure time of 118ms and a gain of 8. Krt20-Alexa 647, Muc2-Alexa 647 and Ki67-Alexa647 signal of all the samples was acquired with the Cy5 filter with an exposure time of 118ms and a gain of 6. All images were visualized with a gamma correction set at 1.0 and the contrast set at 112% in the image controls panel of the NDPview + 2.5.19 or NDP.view 2 U123888-01 software (Hamamatsu, Photonics, France). Dual immunofluorescences were manually quantified on Nanozoomer scanned slides. The results of the analysis are summarized in Table 4.

Table 4: Number of tumors and Tdtomato+ clones assessed by their KRT20+ / MUC2+ status in each timepoint:

Time (days)	Number of tumors	Number of clones assessed for KRT20+	Number of clones assessed for MUC2+
14	3	872	387
28	8	372	611
56	8	69	130

KI67+ on LGR5-EGFP tumors was performed on a total number of: n=2749 Lgr5+ cells, 1798 Lgr5- cells.

Means and confidence intervals for the fraction of cells containing the differentiation markers were computed with a generalized linear model with mixed effects and binomial family. The technical variables xenograft and clone were taken as random effects and the function glmer from the R package "lme4" (20) was used to fit the model, compute p-values and estimate fixed effects. The confint function was used to compute confidence intervals for these estimates.

Quantification of LGR5-EGFP+ cells within the xenographs:

EGFP immunofluorescence intensity was quantified on Nanozoomer scanned slides using the Histogram function of the Fiji software (21). Background noise was set using the mean of several hand-picked background areas within each quantified xeno. Total quantified numbers were: PDO7#1: 4 xenos, PDO7#1 EGFP+ TIC derived: 4 xenos, PDO7#1 EGFP- TIC derived: 3 xenos.

3D reconstruction of 1 month grown LGR5 derived clones

26 paraffin embedded serial sections (3 µm thick) of 1 month induced PDO7#1 LGR5-CreERT2 subcutaneous xenograft were stained for Tdtomato as described above and scanned using NanoZoomer 2.0HT (Hamamatsu) digital scanner at 20X magnification. Regions of interest were cropped at 5x magnification and transformed to .tiff images with the NDPtoolkit software (Hamamatsu). Therefore images were aligned using the Register Virtual Stack Slices plugging of Fiji software (21). Then hyperstack images were cropped, converted to 8-bit grayscale and inverted. A manually trained machine learning pixel classifier (Ilastik software, [17]) was used to create a 3D binary mask and the clones were identified as 3D connected particles. This mask was post processed to discard spurious particles by considering that a valid clone must span at least 2 consecutive sections and a minimum volume of 400 μm^3. The filtered binary mask was overlaid over a volume rendering of the original stack to visualize the clones in context (Imaris 3D software, [22]).

Supplementary References:

1. Doench JG, Hartenian E, Graham DB, Tothova Z, Hegde M, Smith I, Sullender M2, Ebert BL, Xavier RJ, Root DE. (2014) Rational design of highly active sgRNAs for CRISPR-Cas9-mediated gene inactivation. *Nat Biotechnol* 32(12):1262-7

2. Calon A, Lonardo E, Berenguer-Llergo A, Espinet E, Hernando-Momblona X, Iglesias M, Sevillano M, Palomo-Ponce S, Tauriello DV, Byrom D, Cortina C, Morral C, Barceló C, Tosi S, Riera A, Attolini CS, Rossell D, Sancho E, Batlle E (2015) Stromal gene expression defines por-prognosis subtype in colorectal cancer. *Nat Genet* 47(4):320-9.

3. Merlos-Suárez A, Barriga FM, Jung P, Iglesias M, Céspedes MV, Rossell D, Sevillano M, Hernando-Momblona X, da Silva-Diz V, Muñoz P, Clevers H, Sancho E, Mangues R, Batlle E. (2011) The intestinal stem cell signature identifies colorectal cancer stem cells and predicts disease relapse. *Cell Stem Cell* 8(5): 511-24

4. Gonzalez-Roca E, Garcia-Albéniz X, Rodriguez-Mulero S, Gomis RR, Kornacker K, Auer H. (2010). Accurate expression profiling of very small cell populations. *PLoS One* 5(12):e14418.

5. Irizarry RA, Hobbs B, Collin F, Beazer-Barclay YD, Antonellis KJ, Scherf U, Speed TP. (2003) Exploration, normalization, and summaries of high density oligonucleotide array probe level data. *Biostatistics* 4(2):249-64.

6. Bolstad BM. (2004). Low Level Analysis of High-density Oligonucleotide Array Data: Background, Normalization and Summarization. PhD thesis, University of California, Berkeley.

7. Affymetrix Analysis Center. Netaffx. https://www.affymetrix.com/analysis/index.affx.

8. Eklund AC, Szallasi Z. (2008) Correction of technical bias in clinical microarray data improves concordance with known biological information. *Genome Biol* 9(2):R26.

9. Ritchie ME, Phipson B, Wu D, Hu Y, Law CW, Shi W and Smyth GK (2015). "limma powers differential expression analyses for RNA-sequencing and microarray studies." *Nucleic Acids Research* 43(7), pp. e47.

10. Subramanian, A. Gene set enrichment analysis: A knowledge-based approach for interpreting genome-wide expression profiles. (2005) *PNAS* 102(43):15545–15550

11. Muñoz J, Stange DE, Schepers AG, van de Wetering M, Koo BK, Itzkovitz S, Volckmann R, Kung KS, Koster J, Radulescu S, Myant K, Versteeg R, Sansom OJ, van Es JH, Barker N, van Oudenaarden A, Mohammed S, Heck AJ, Clevers H. (2012) The Lgr5 intestinal stem cell signature: robust expression of proposed quiescent '+4' cell markers. *EMBO J*. 31(14):3079-91

12. Basak O, van de Born M, Korving J, Beumer J, van der Elst S, van Es JH, Clevers H (2014) Mapping early fate determination in Lgr5+ crypt stem cells using a novel Ki67-RFP allele. EMBO J 33: 2057-68

13. Li H. and Durbin R. (2009) Fast and accurate short read alignment with Burrows-Wheeler Transform. *Bioinformatics* 25:1754-60.

14. McKenna A, Hanna M, Banks E, Sivachenko A, Cibulskis K, Kernytsky A, Garimella K, Altshuler D, Gabriel S, Daly M, DePristo MA (2010) The Genome Analysis Toolkit: a MapReduce framework for analyzing next-generation DNA sequencing data. *Genome Research* 20:1297-303

15. Koboldt DC, Zhang Q, Larson DE, Shen D, McLellan MD, Lin L, Miller CA, Mardis ER, Ding L, Wilson RK. (2012) VarScan 2: somatic mutation and copy number alteration discovery in cancer by exome sequencing. *Genome Research*. 22(3):568-76.

16. Witten, I.H., Frank, E., Trigg, L., Hall, M., Holmes, G. & Cunningham, S.J. (1999) Weka: practical machine learning tools and techniques with Java implementations. *Proceedings of the ICONIP/ANZIIS/ANNES'99 Workshop on Emerging Knowledge Engineering and Connectionist-Based Information Systems* 192–196. DOI 10.5281/zenodo.59290

17. C.Sommer, C.Strähle, U.Köthe, F.A.Hamprecht (2011) *Eighth IEEE International Symposium on Biomedical Imaging (ISBI). Proceedings,* 230-233

18. Chris Fraley. Et al. (2012) mclust Version 4 for R: Normal Mixture Modeling for Model-Based Clustering, Classification, and Density Estimation Technical Report No. 597, Department of Statistics, University of Washington

19. R Development Core Team (2008). R: A language and environment for statistical computing. R Foundation for Statistical Computing, Vienna, Austria. ISBN 3-900051-07-0

20. Douglas Bates, Martin Maechler, Ben Bolker, Steve Walker (2015). Fitting Linear Mixed-Effects Models Using lme4. *Journal of Statistical Software* 67(1), 1-48.

21.Schindelin J, Arganda-Carreras I, Frise E, et al. (2012). Fiji: an open-source platform for biological-image analysis. *Nat Methods*. 9(7):676-682.
