# Supplementary material for: A genome editing approach to study cancer stem cells in human tumors
Source: EMBO Mol Med. 2017 May 3;9(7):869–79. doi: 10.15252/emmm.201707550 (PMC5494503; doi:10.15252/emmm.201707550)
Supplement: Supplementary file 2 — Expanded View Figures PDF [file EMMM-9-869-s002.pdf]

Expanded View Figures

Figure EV1. Characterization of LGR5-EGFP^{+} cells in PDO#6.

A Flow cytometry profiles at day 20 post-nucleofection.
B Live imaging of PDO#6 LGR5-EGFP#1 knock-ins. Scale bars indicate 50 μm.
C FACS profiles showing EGFP-high (green), EGFP-low (blue) and EGFP-negative (gray) cells from dissociated PDO#6-LGR5-EGFP#1 organoids.
D Relative mRNA expression (RT–qPCR) of key marker genes of the cell fractions mentioned in (C). Values show mean ± standard deviation (s.d.) of three measurements.
E Representative images of EGFP patterns analyzed by immunofluorescence on a section of PDO#6-LGR5-EGFP#1-derived subcutaneous xenograft. White squares indicate position of the insets in the xenograft. Scale bars indicate 1 mm for low magnification picture and 100 μm for insets.
F Dual immunofluorescence for KRT20 and LGR5 demonstrating complementary expression domains of the two marker genes. Dashed line marks expression domains of adjacent glands. Scale bar indicates 100 μm.
G Dual immunofluorescence on paraffin sections of clone #1. White arrows point to LGR5^{-}/MUC2^{+} cells. Scale bar indicates 100 μm.
H Flow cytometry analysis of disaggregated xenografts generated by PDO#6-LGR5-EGFP#2.
I Relative expression level by RT–qPCR of intestinal stem and differentiation genes in EGFP^{+} versus EGFP^{-} (EPCAM^{+}) cells isolated from disaggregated xenografts. Values show mean ± s.d. of three measurements.
J Representative pictures and quantifications of organoid formation generated by EGFP^{+} and EGFP^{-} cells isolated from xenografts. Scale bars indicate 1 mm. ($n = 4$ wells per condition). Data is represented as mean ± s.d. Differences were assessed with Student's *t*-test: ****P*-value < 0.005. The exact *P*-values are specified in Appendix Table S5.
K Representative flow cytometry analysis of 15 days grown organoids from the EGFP^{+} and EGFP^{-} sorted populations.

© 2017 The Authors

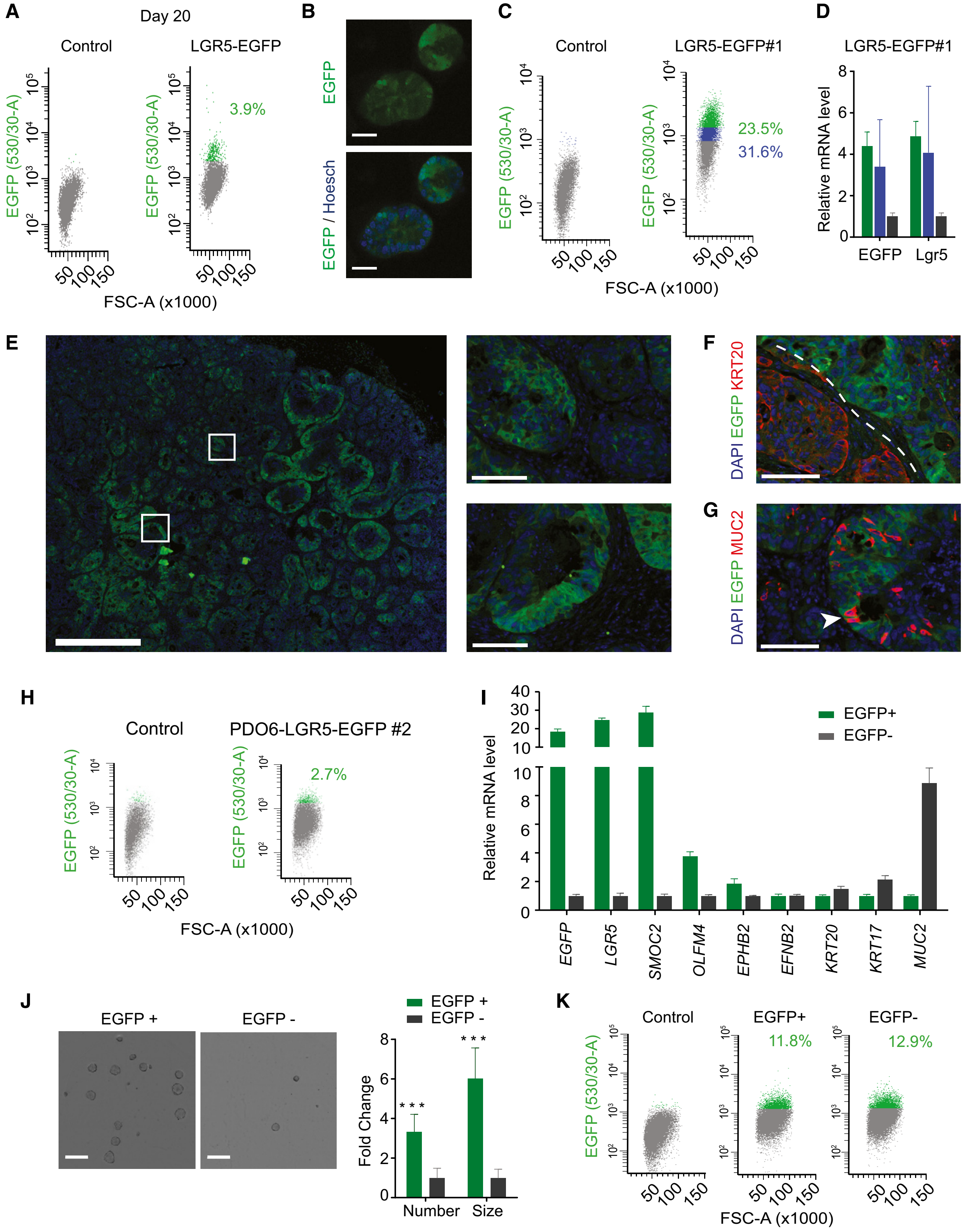
A
Day 20
Control
LGR5-EGFP
3.9%
EGFP (530/30-A)
FSC-A (x1000)
B
EGFP
EGFP / Hoesch
C
Control
LGR5-EGFP#1
23.5%
31.6%
EGFP (530/30-A)
FSC-A (x1000)
D
LGR5-EGFP#1
Relative mRNA level
EGFP
Lgr5
E
F
DAPI EGFP KRT20
G
DAPI EGFP MUC2
H
Control
PDO6-LGR5-EGFP #2
2.7%
EGFP (530/30-A)
FSC-A (x1000)
I
EGFP+
EGFP-
Relative mRNA level
EGFP
LGR5
SMOC2
OLFM4
EPHB2
EFNB2
KRT20
KRT17
MUC2
J
EGFP +
EGFP -
Fold Change
Number
Size
K
Control
EGFP+
EGFP-
11.8%
12.9%
EGFP (530/30-A)
FSC-A (x1000)

Figure EV1.

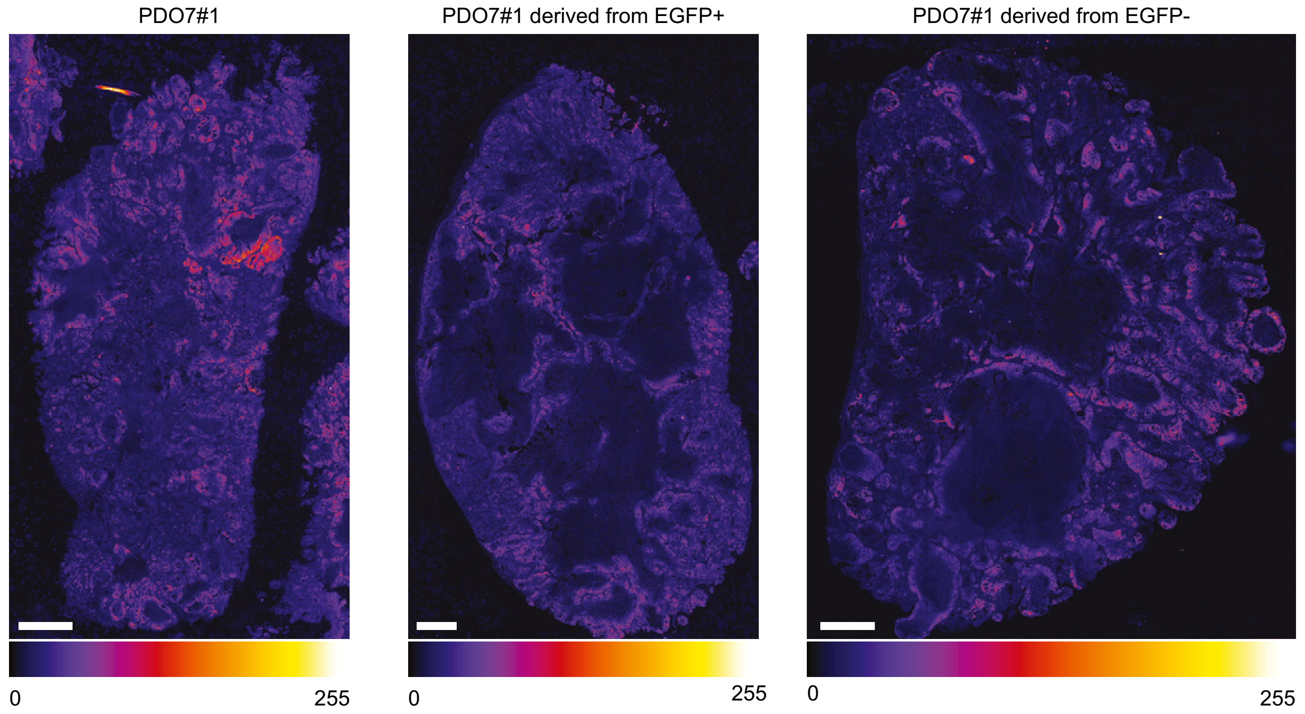

Figure EV2. Subcutaneous xenografts exhibit heterogeneous LGR5 levels.
EGFP immunofluorescence on whole tumors formed by PDO7#1 or by $EGFP^+$ and $EGFP^-$ cells in tumor initiation experiments. They are representative of the images used for histogram intensity plotting in Fig 2. The intensity of the signal is indicated by the lookup table (gray scale intensity of 256 values in 8 bit per pixel images). Scale bars indicate 1 mm.

 © 2017 The Authors

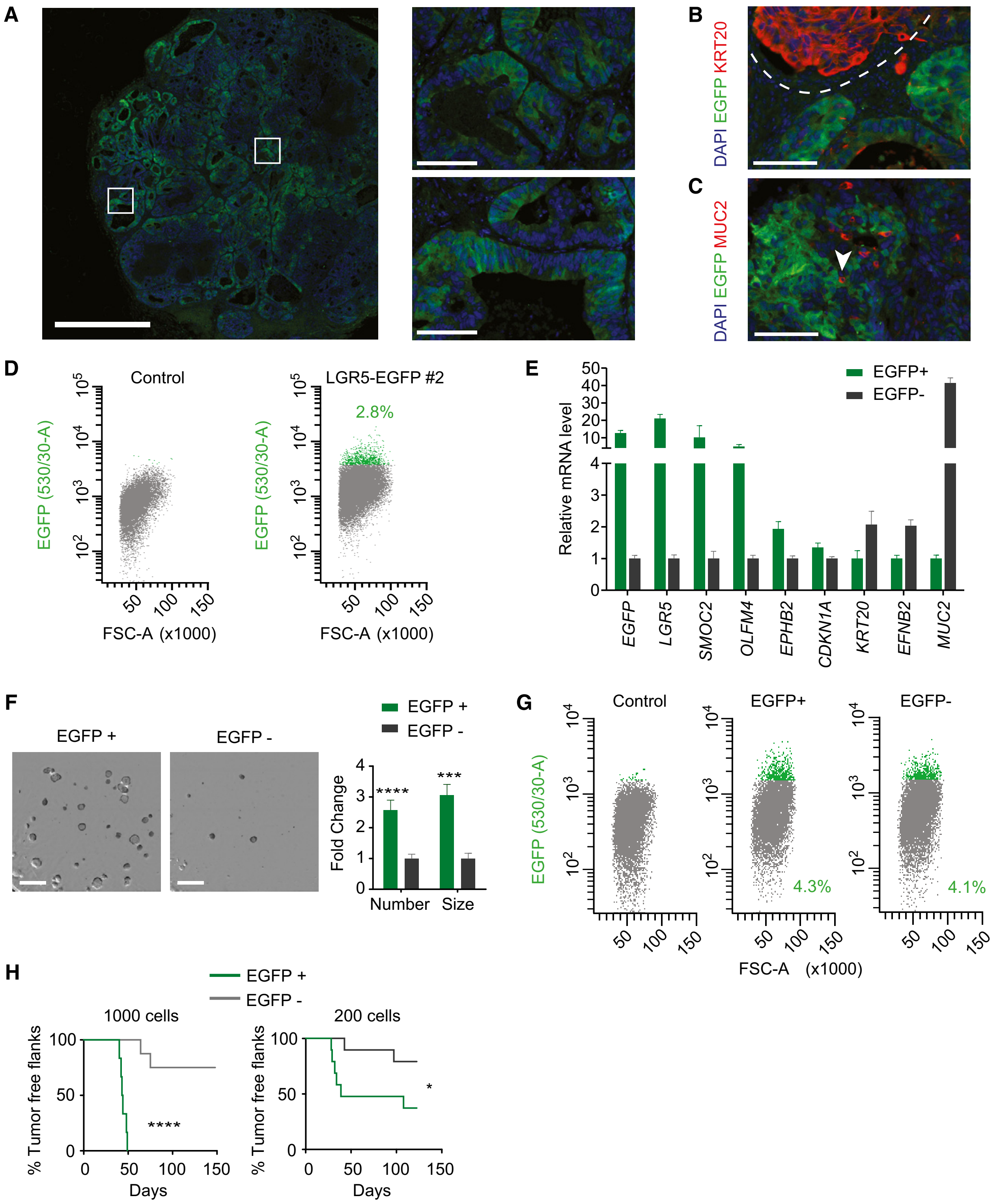
A
B
DAPI EGFP KRT20
C
DAPI EGFP MUC2
D
Control
LGR5-EGFP #2
2.8%
EGFP (530/30-A)
FSC-A (x1000)
E
Relative mRNA level
EGFP+
EGFP-
EGFP
LGR5
SMOC2
OLFM4
EPHB2
CDKN1A
KRT20
EFNB2
MUC2
F
EGFP +
EGFP -
Fold Change
****
***
Number
Size
G
Control
EGFP+
EGFP-
4.3%
4.1%
FSC-A (x1000)
H
1000 cells
200 cells
% Tumor free flanks
Days
****
*

Figure EV3. Characterization of LGR5-EGFP$^+$ cells in clone #2 of PDO#7-LGR5-EGFP.

A Representative images of EGFP patterns analyzed by immunofluorescence on a section of PDO#7-LGR5-EGFP#2-derived subcutaneous xenograft. White squares are magnified in insets. Scale bars indicate 1 mm for the whole xenograft, 100 μm for the magnified insets.
B Dual immunofluorescence of clone #2 for KRT20 and LGR5-EGFP that illustrates complementary expression domains of the marker genes. Dashed line delimits expression domains in adjacent glands. Scale bar indicates 100 μm.
C Dual immunofluorescence of clone #2 for MUC2 and LGR5-EGFP. White arrows point to LGR5$^-$/MUC2$^+$ tumor cells. Scale bar indicates 100 μm.
D Flow cytometry profiles of EGFP$^+$ and EGFP$^-$ disaggregated xenografts. Only EPCAM$^+$ cells are shown.
E Relative expression levels assessed by RT–qPCR of ISC and differentiation genes in EGFP$^+$ and EGFP$^-$ cells isolated from disaggregated xenografts. Values show mean ± standard deviation (s.d.) of three measurements.
F Representative images and quantifications of organoid formation by EGFP$^+$ versus EGFP$^-$ cells isolated from xenografts (n = 4 wells per condition). Data is represented as mean ± s.d. Scale bars indicate 1 mm.
G Representative flow cytometry analysis of 15-day grown organoids formed by EGFP$^+$ and EGFP$^-$ sorted populations.
H Tumor initiation capacity of 1,000 and 200 sorted epithelial cells from xenografts that were re-inoculated into recipient mice. Graphs show Kaplan–Meier plots (n = 9 xenografts for condition. In EGFP$^+$ 1,000 cells n = 6).

Data information: Differences in organoid formation were assessed with Student's t-test and by log-rank (Mantel–Cox) test in tumor initiation assays: *P-value < 0.05, ***P-value < 0.005, ****P-value < 0.0001. The exact P-values are specified in Appendix Table S5.

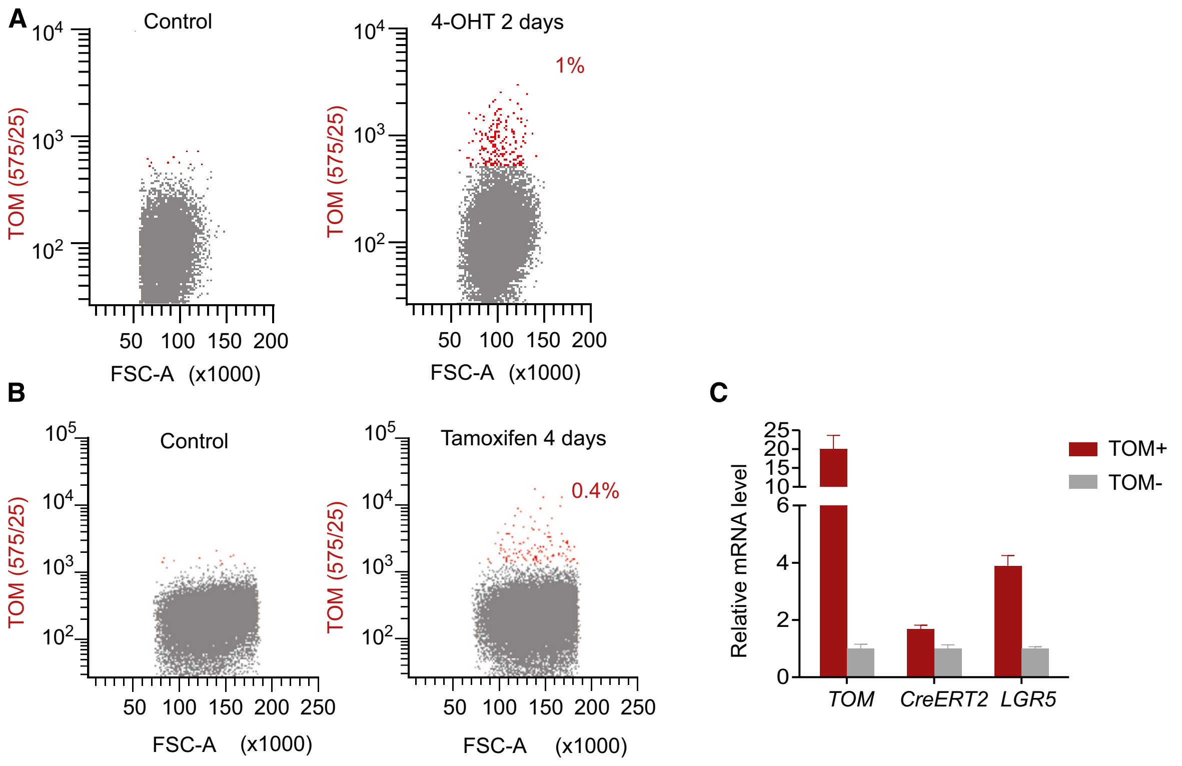

Figure EV4. TOM$^+$ cells express LGR5 shortly after recombination.

A Flow cytometry analysis of PDO#7 AAVS1-LSL-TOM LGR5-CreERT2#1 treated for 2 days with 1 μM 4-hydroxytamoxifen (4-OHT).
B Flow cytometry profiles of PDO#7 AAVS1-LSL-TOM LGR5-CreERT2#1-derived xenograft 4 days after injection of tamoxifen.
C Relative mRNA expression levels of LGR5 in TOM$^+$ versus TOM$^-$ populations by RT-qPCR. Values show mean ± standard deviation of three measurements.

 © 2017 The Authors

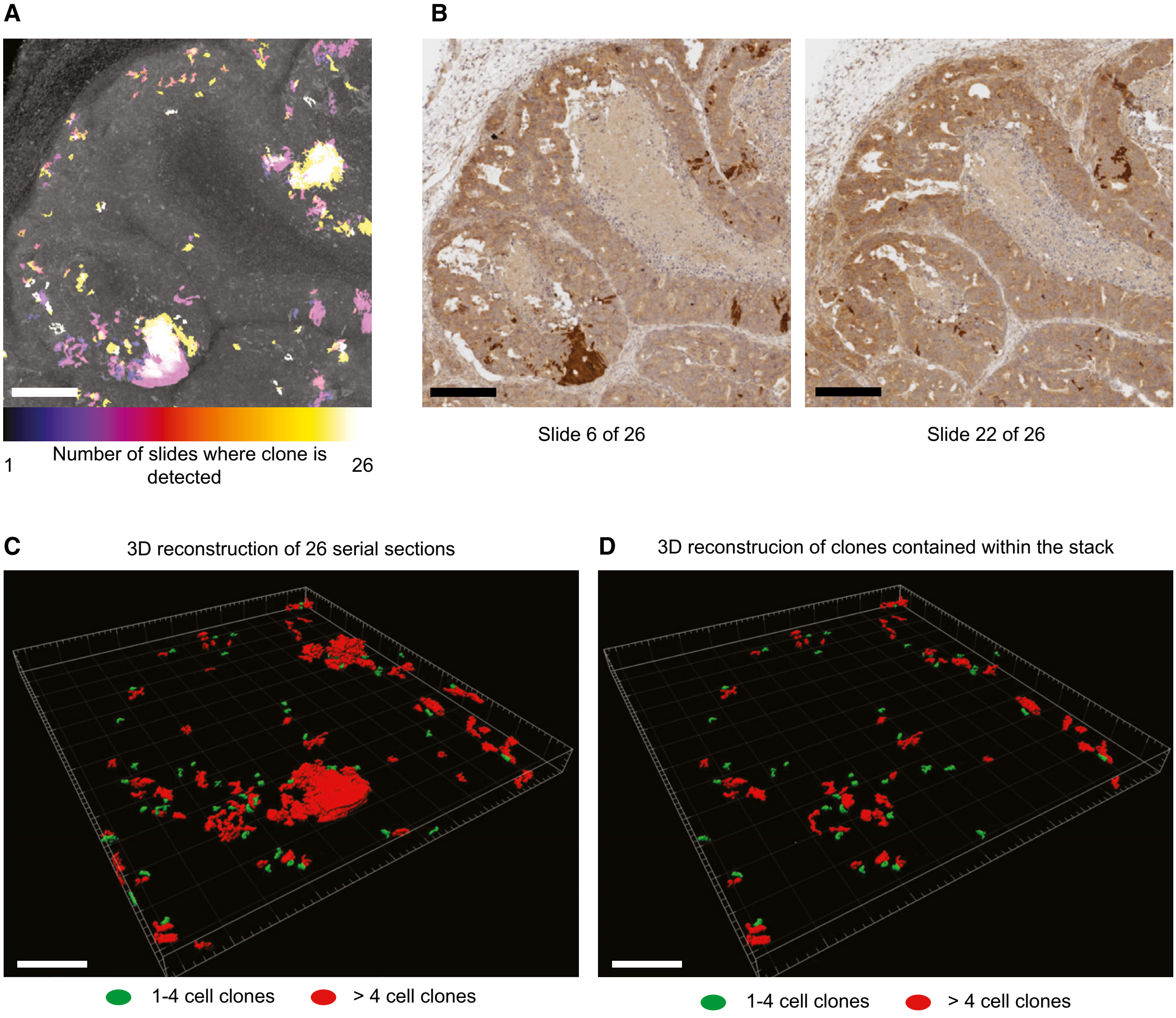

Figure EV5. 3D reconstruction of 1-month grown LGR5 clones.

A *Z*-projection of clones in 26 serial sections of subcutaneous PDO#7 LGR5-CreERT2#1 xenografts 1 month after tamoxifen injection. Color scale denotes the number of sections occupied by a given clone. Scale bar is 200 μm.

B Example of two slides stained for Tomato used for 3D reconstruction. Scale bars are 200 μm.

C Projection of the 3D reconstructed clones. Scale bar indicates 200 μm.

D Projection of the 3D reconstructed clones that do not contact borders and therefore are fully embedded within the *Z* plan analyzed. Scale bar indicates 200 μm.

© 2017 The Authors
