# Supplementary figures and images for: A genome editing approach to study cancer stem cells in human tumors
Source: EMBO Mol Med. 2017 May 3;9(7):869–79. doi: 10.15252/emmm.201707550 (PMC5494503; doi:10.15252/emmm.201707550)

B

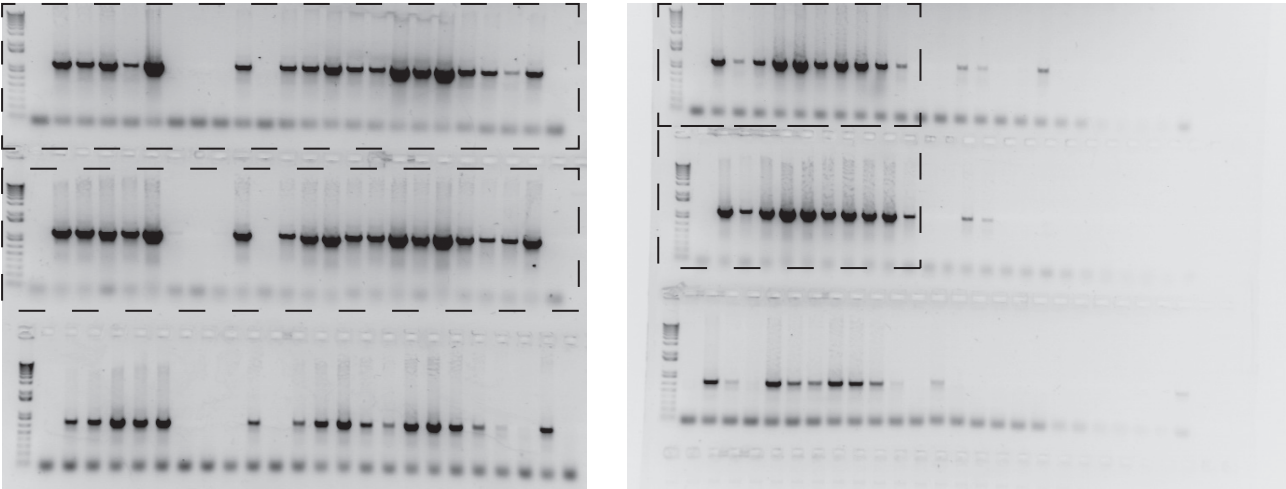

C

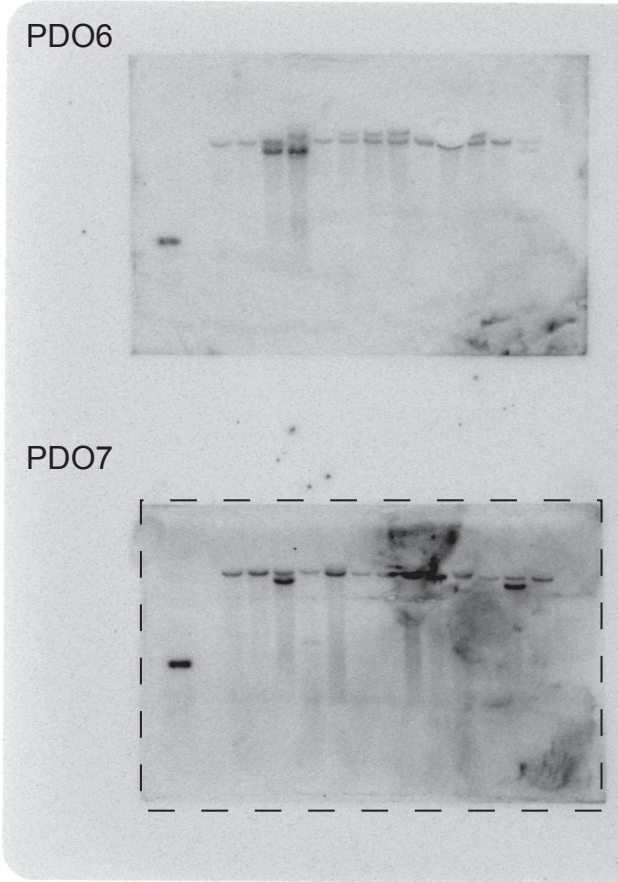

Supplement: Supplementary file 4 — Source Data for Appendix [file EMMM-9-869-s004.zip › SourceData_Appendix_Fig_S1.pdf]
